# Differentially Expressed Genes Associated With Prognosis in Locally Advanced Lymph Node-Negative Prostate Cancer

**DOI:** 10.3389/fgene.2019.00730

**Published:** 2019-08-09

**Authors:** Elena A. Pudova, Elena N. Lukyanova, Kirill M. Nyushko, Dmitry S. Mikhaylenko, Andrew R. Zaretsky, Anastasiya V. Snezhkina, Maria V. Savvateeva, Anastasiya A. Kobelyatskaya, Nataliya V. Melnikova, Nadezhda N. Volchenko, Gennady D. Efremov, Kseniya M. Klimina, Anastasiya A. Belova, Marina V. Kiseleva, Andrey D. Kaprin, Boris Y. Alekseev, George S. Krasnov, Anna V. Kudryavtseva

**Affiliations:** ^1^Engelhardt Institute of Molecular Biology, Russian Academy of Sciences, Moscow, Russia; ^2^National Medical Research Radiological Center, Ministry of Health of the Russian Federation, Moscow, Russia; ^3^Federal State Autonomous Educational Institution of Higher Education, I.M. Sechenov First Moscow State Medical University of the Ministry of Health of the Russian Federation, Moscow, Russia; ^4^Vavilov Institute of General Genetics, Russian Academy of Sciences, Moscow, Russia

**Keywords:** locally advanced prostate cancer, prognostic markers, TCGA, RNA-Seq, TMPRSS2-ERG

## Abstract

Older age is one of the main risk factors for cancer development. The incidence of prostate cancer, as a multifactorial disease, also depends upon demographic factors, race, and genetic predisposition. Prostate cancer most frequently occurs in men over 60 years of age, indicating a clear association between older age and disease onset. Carcinogenesis is followed by the deregulation of many genes, and some of these changes could serve as biomarkers for diagnosis, prognosis, prediction of drug therapy efficacy, as well as possible therapeutic targets. We have performed a bioinformatic analysis of a The Cancer Genome Atlas (TCGA) data and RNA-Seq profiling of a Russian patient cohort to reveal prognostic markers of locally advanced lymph node-negative prostate cancer (lymph node-negative LAPC). We also aimed to identify markers of the most common molecular subtype of prostate cancer carrying a fusion transcript *TMPRSS2-ERG*. We have found several genes that were differently expressed between the favorable and unfavorable prognosis groups and involved in the enriched KEGG pathways based on the TCGA (*B4GALNT4*, *PTK6*, and *CHAT*) and Russian patient cohort data (*AKR1C1* and *AKR1C3*). Additionally, we revealed such genes for the *TMPRSS2-ERG* prostate cancer molecular subtype (*B4GALNT4*, *ASRGL1*, *MYBPC1*, *RGS11*, *SLC6A14*, *GALNT13*, and *ST6GALNAC1*). Obtained results contribute to a better understanding of the molecular mechanisms behind prostate cancer progression and could be used for further development of the LAPC prognosis marker panel.

## Introduction

Aging is a complex phenomenon, which is characterized by the deregulation of various cellular processes and involves metabolic, epigenomic, transcriptomic, genomic, and proteomic alterations, and ultimately leads to death. Aberrant gene expression, somatic mutations, DNA damage caused by exposure to ionizing radiation, reactive oxygen species, and chemicals accumulate throughout the life of a cell. These alterations can initiate activation of oncogenes and deactivation of tumor suppressor genes resulting in tumorigenesis. Moreover, one of the main causes of cancers is the disruption of cellular senescence, which is associated with proliferative arrest blocking multiplication of potentially malignant cells. The association between aging and cancer is well documented in the literature, and several cancer types are classified as age-related diseases (Kudryavtseva et al., 2016).

Being a multifactorial age-related disease, prostate cancer (PC) also depends upon demographic factors, race, and genetic predisposition (Belova et al., 2017). A clear association between older age and prostate cancer is confirmed by the elevation of incidence in men over 60 years of age (Vinjamoori et al., 2012). PC is associated with aging through the disruption of glandular homeostasis, which is caused by an imbalance between cell proliferation and death. The imbalance between mitosis and apoptosis also leads to various diseases, including prostate cancer (Plati et al., 2011).

Carcinogenesis is associated with the disruption in the functioning of many genes that lead to deregulations of metabolic and signaling pathways (Cherkasova et al., 2011; Oparina et al., 2013; Fedorova et al., 2015; Snezhkina et al., 2016a; Fedorova et al., 2017; Kudryavtseva et al., 2018; Snezhkina et al., 2018a; Pudova et al., 2018). Some of these changes can serve as markers for diagnosis and prognosis, predicting the efficacy of drug therapy (Krasnov et al., 2015; Knyazev et al., 2016; Jhun et al., 2017; Kudryavtseva et al., 2019), as well as possible therapeutic targets. However, a large number of studies indicate difficulties in the choice of universal prognostic markers (Dmitriev et al., 2015) that are equally effective for various stages of the disease. Moreover, most cancer types and histological subgroups of cancer are divided into subtypes. These tumor subtypes differ in clinical and molecular genetic characteristics, and each could be identified using a set of markers that predict the aggressiveness of the disease and evaluate sensitivity to medicines (Dmitriev et al., 2014; Krasnov et al., 2015; Snezhkina et al., 2016b; Krasnov et al., 2017; Snezhkina et al., 2018b).

Therefore, a detailed study of the molecular genetic characteristics of different PC subgroups, as well as the selection of markers for each molecular subtype and propensity for metastasis, is particularly important.

Based on the data from “The Cancer Genome Atlas” (TCGA, https://portal.gdc.cancer.gov) project, several molecular subtypes of PC have been identified. The most common ones comprise three groups characterized by the formation of the fusion transcripts (*TMPRSS2-ERG*, *TMPRSS2-ETV1*, and *TMPRSS2-ETV4*) and a group that has somatic mutations in the *SPOP* gene (The Cancer Genome Atlas Research Network, 2015). Making well informed decisions on the applicability of adjuvant therapy following radical PC surgical treatment remains an unanswered problem. This problem is relevant for cohorts of patients with locally advanced prostate cancer (LAPC) without lymphatic dissemination (LD), and particularly for those patients with the most widely represented molecular subtype of PC associated with the expression of the fusion transcript *TMPRSS2-ERG* (approximately 40% of PC patients). Many researchers attribute this transcript to factors associated with unfavorable disease prognosis. One of the most effective approaches for identification of prognostic markers is to use transcriptome profiling data deposited in the TCGA consortium database (approximately 500 prostate cancer samples) (The Cancer Genome Atlas Research Network, 2015).

Currently, there are several commercially available services that help to predict the aggressiveness of the disease and to choose optimal treatment strategies. However, these decision-making gene expression panels were initially developed and validated on patient cohorts with a significantly different ethnic composition to that of Russian patients.

Thus, the aim of this study was to identify reliable prognostic markers of both locally advanced lymph node-negative prostate cancer and the most common PC molecular subtype, *TMPRSS2-ERG*.

## Materials and Methods

### Tumor Samples

Prostate cancer samples and adjacent histologically normal tissues were obtained from patients that underwent surgical intervention in the P.A. Hertzen Moscow Oncology Research Center (branch of the National Medical Research Radiological Center, Ministry of Health of the Russian Federation) in 2015–2016. All materials were collected and characterized by the Organization’s Pathology Department according to the WHO Classification of Tumours of the Urinary System and Male Genital Organs (Moch et al., 2016). Each sample contained a minimum 70% of tumor cells. Tissue samples were immediately frozen and stored in liquid nitrogen following surgical resection.

In the current study, we used 32 lymph node-negative LAPC (adenocarcinoma) samples obtained from patients that underwent surgical treatment and did not receive neoadjuvant therapy. The samples have the following characteristics: pT3a and pT3b categories, negative resection margins, any PSA value, and any Gleason score (**Supplementary Table S1**). We did not include normal prostate tissues in the current study.

All patients have had at least 1 year of follow-up. The serum level of PSA was recorded every 6 months. Biochemical reoccurrence of the disease was determined as an increase in PSA level > 0.2 ng/ml in three consecutive measurements with an interval of not less than 2 weeks. Based on these data, patients were divided into the favorable prognosis group (no relapse within a year) and unfavorable prognosis group (biochemical relapse within a year).

The study was approved by The Ethics committee of P.A. Hertzen Moscow Oncology Research Center, Ministry of Health of the Russian Federation. The study was done in accordance with the principles outlined in the Declaration of Helsinki (1964). All patients gave their informed consent for participation in the study.

### Isolation of RNA, Library Preparation, and Next Generation Sequencing (NGS)

Total RNA was isolated from 32 tumor samples using a MagNA Pure Compact RNA Isolation Kit (Roche, Switzerland). The RNA concentration was determined using a Qubit 2.0 Fluorometer (Thermo Fisher Scientific, USA). The RIN (RNA Integrity Number) parameter, which characterizes the integrity of RNA, was evaluated using an Agilent 2100 Bioanalyzer (Agilent Technologies, USA). RIN for all samples studied was no less than 8.0.

Libraries were prepared using a TruSeq^®^ Stranded mRNA LT Kit (Illumina, USA) according to the manufacturer’s protocol. The average size of prepared cDNA libraries was 300 bp. Libraries were validated prior to sequencing using a quantitative PCR (qPCR).

Sequencing was performed on a NextSeq500 System (Illumina) at the EIMB RAS “Genome” Center [http://www.eimb.ru/rus/ckp/ccu_genome_c.php]. Read length was 75 bp (single-end mode).

### RNA-Seq Data Analysis of the Russian Patient Cohort

Read quality was evaluated using FastqQC [http://www.bioinformatics.babraham.ac.uk/publications.html]. Read pseudo-alignment to the reference genome (assembly GRCh38) and their counting were performed using Kallisto (Bray et al., 2016) [https://pachterlab.github.io/kallisto/]. The read count table was exported and used in the statistical environment R [https://www.r-project.org/]. To analyze the differential expression of genes, the Bioconductor package DESeq2 (Love et al., 2014) [http://www.bioconductor.org/packages/release/bioc/html/DESeq2.html] was used. Gene expression-level differences were accepted as statistically significant if they underwent a 2-fold change or greater and had a *p*-value of less than 0.05. Tumor tissue samples demonstrate heterogeneous gene expression profiles that do not have Gaussian or any other parametric distribution across the cohort. Hence, standard edgeR or DESeq2 tests should be used followed with a non-parametric test (e.g., Mann–Whitney *U* test). The decision should be made after the mandatory manual examination of expression profiles across the sampling. In the study, we supplied our data with Mann–Whitney *p*-values (the lowest rank was assigned to all samples with CPM [read counts per million] less than 0.5). Differences in gene expression levels (fold changes) between the favorable and unfavorable prognosis groups were transformed into the binary logarithms of fold changes (LogFC). Gene Ontology and KEGG pathway enrichment analysis were done using the clusterProfiler Bioconductor package (Yu et al., 2012) [http://bioconductor.org/packages/release/bioc/html/clusterProfiler.html].

### Bioinformatic Analysis of the TCGA Data

To identify transcriptomic markers of unfavorable prognosis, the bioinformatic analysis was carried out using the results of TCGA transcriptome sequencing data generated on an Illumina platform (HiSeq 1500 System) and deposited into the TCGA database. We focused on the study of Caucasian patients, as this cohort is closest to the Russian population (Caucasians are identified in the database as “white”). The total sample size was 134 individuals. Next, we isolated a group of patients with locally advanced lymph node-negative prostate cancer according to the pathologic classification (pTNM). We only included patients that had undergone a prostatectomy, which is generally accompanied by lymphadenectomy (“Nx” was unacceptable). Patients who received neoadjuvant therapy were not included in the study. Finally, in the analysis, we included 113/21 patients with favorable/unfavorable prognosis, among them—42/10 patients of *TMPRSS2-ERG* group.

The transcriptome sequencing data from the TCGA (read counts) were exported for further analysis in R environment with the DESeq2 and clusterProfiler packages as described above.

## Results

### Bioinformatic Analysis of the TCGA Data

**Figure 1:**
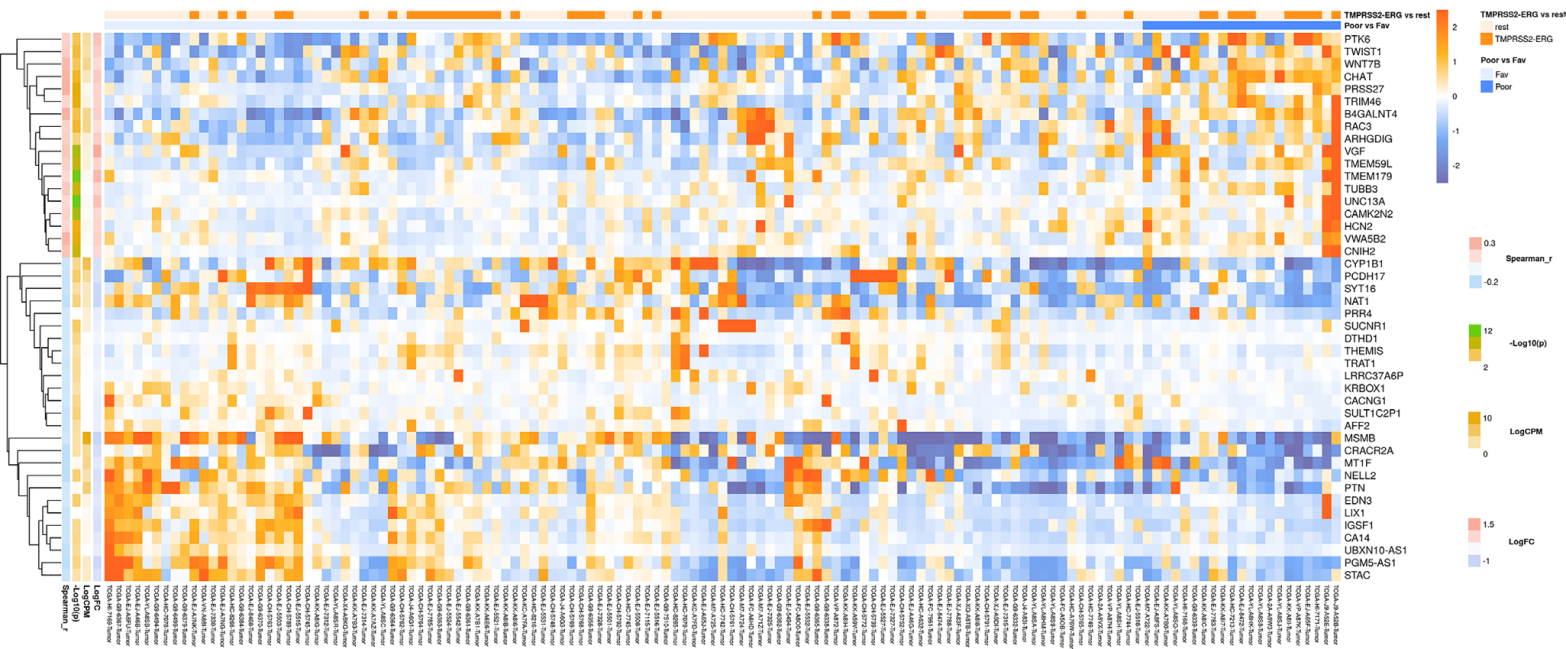
Heatmap demonstrating log relative expression level of top differentially expressed genes between the groups of adverse and favorable prognosis (TCGA data). Cell colors (blue-white-orange gradient) correspond the binary logarithm of the ratio of the expression level in a current sample to the average level across all the samples (per each gene). Blue - expression level is below the average, orange - above the average. Spearman_r—Spearman’s rank correlation coefficient. -Log10(*p*) is the negative logarithm of DESeq’s *p*-value. LogCPM—average binary logarithm of read counts per million (CPM). LogFC—binary logarithm of expression level fold change (samples with adverse prognosis compared to favorable prognosis).

**Figure 2:**
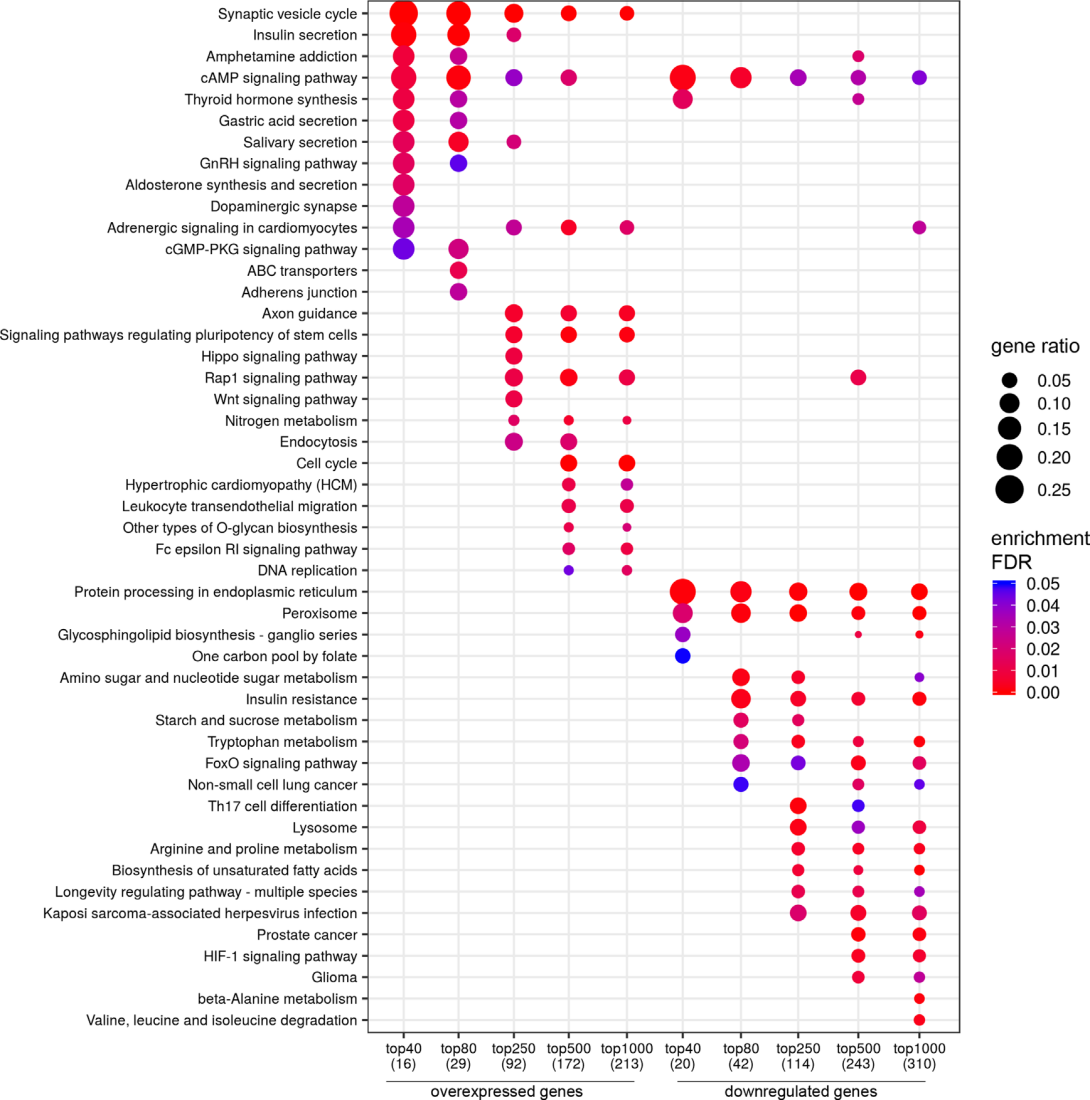
Dotplot showing the results of KEGG pathways enrichment analyses performed for top-40, 80, 250, 500, and 1,000 differentially expressed genes (either overexpressed or downregulated) between the groups of adverse and favorable prognosis (TCGA data). All the genes have passed the following thresholds: DESeq *p*-value < 0.01, Mann–Whitney *p*-value < 0.05, average expression-level fold change > 25%. Dot size indicates *k*/*n* ratio (“gene ratio”), where *k* is the number of genes participating in the current KEGG pathway (within top-40, top-80, or other top lists), and n is the number of genes (also within top-40, top-80, or other top lists) annotated as participants of any KEGG pathway (these numbers are provided in the brackets in the bottom of the figure). Dot color indicates the enrichment test FDR (Fisher’s exact test).

**Figure 3:**
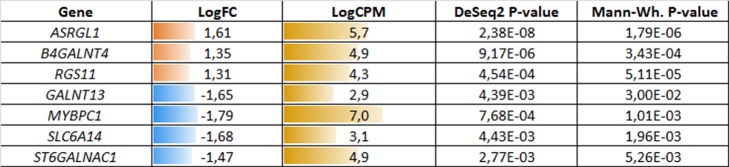
Potential markers of unfavorable prognosis in lymph node-negative locally advanced prostate cancer (LAPC) involved in the enriched KEGG pathways (TCGA data). LogFC—trimmed binary logarithm of average expression level fold change (between the groups of favorable and unfavorable prognosis); LogCPM—binary logarithm of read counts per million (CPM).

For lymph node-negative LAPC samples, a comparison between the favorable (no relapse for the entire observation period) and unfavorable (biochemical reoccurrence) prognosis groups was made, and 173 differentially expressed genes (DEGs) were revealed (**Supplementary Table S2**). This list includes genes for which the expression level underwent a 2-fold or higher changes and that were passed DESeq2 *p* < 0.05 threshold. Using these data, we performed Mann–Whitney test and identified 44 genes with Mann–Whitney *p* < 0.05 that could be potential markers of unfavorable prognosis in lymph node-negative LAPC (**Figure 1**). Next, KEGG pathway enrichment analysis was carried out using all set of identified DEGs (173 genes). This allowed to reveal biological pathways that are most likely responsible for phenotypic and clinical differences between favorable and unfavorable prognosis groups (**Figure 2**, **Supplementary Table S5**). Three genes (*B4GALNT4*, *CHAT*, and *PTK6*) with Mann–Whitney *p* < 0.05 were involved in the enriched KEGG pathways (**Figure 3**).

**Figure 4:**
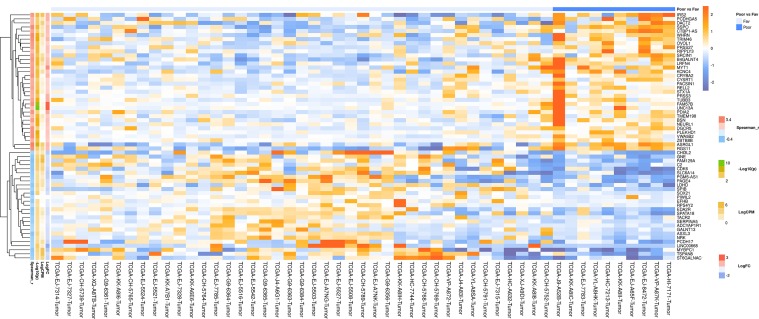
Heatmap demonstrating log relative expression level of top differentially expressed genes between the groups of adverse and favorable prognosis (TCGA data; TMPRSS2-ERG-positive samples). Cell colors (blue-white-orange gradient) correspond the binary logarithm of the ratio of the expression level in a current sample to the average level across all the samples (per each gene). Blue - expression level is below the average, orange - above the average. Spearman_r—Spearman’s rank correlation coefficient. -Log10(p) is the negative logarithm of DESeq’s *p*-value. LogCPM—average binary logarithm of read counts per million (CPM). LogFC—binary logarithm of expression-level fold change (sample with adverse prognosis compared to favorable prognosis).

**Figure 5:**
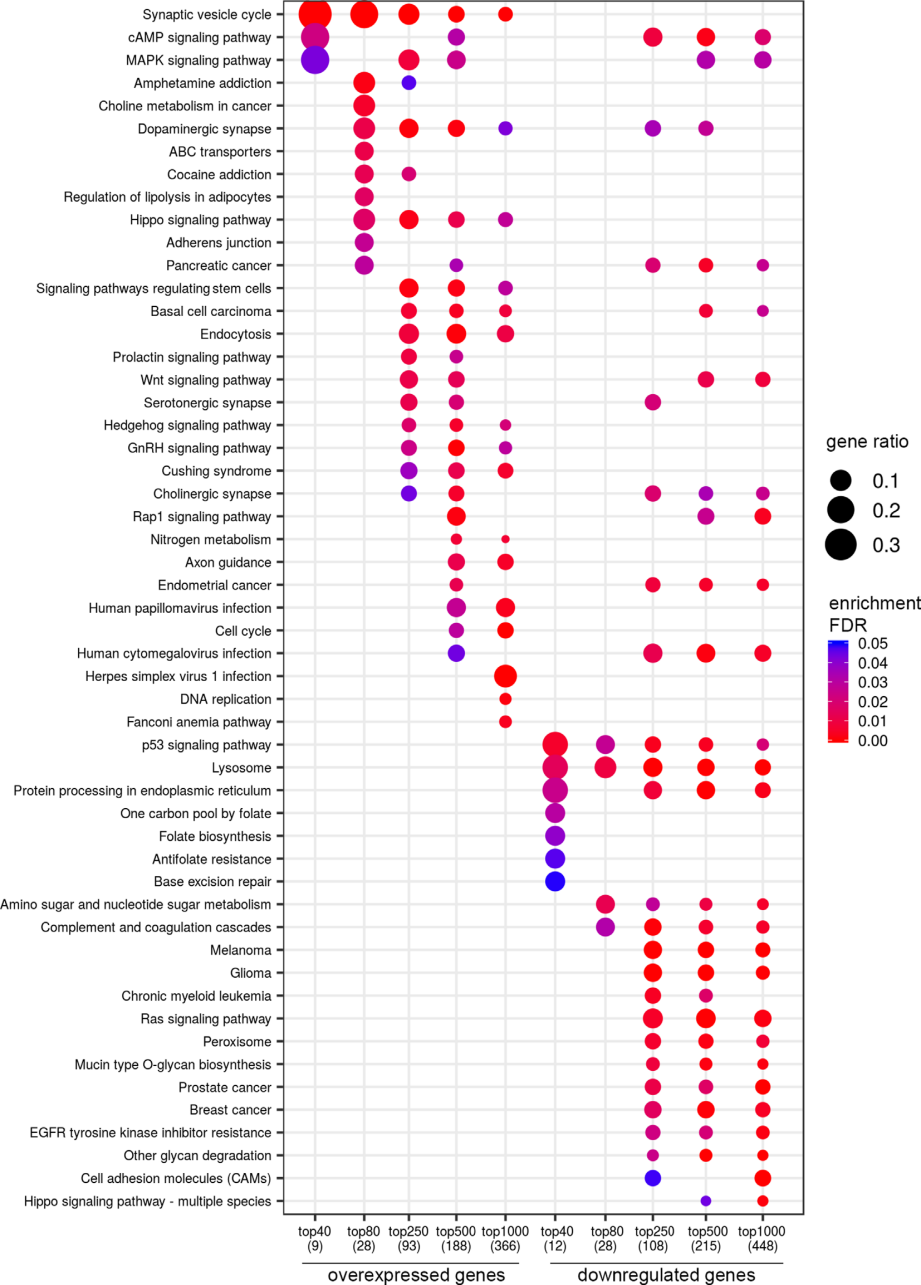
Dotplot showing the results of KEGG pathways enrichment analyses performed for top-40, 80, 250, 500, and 1,000 differentially expressed genes (either overexpressed or downregulated) between the groups of adverse and favorable prognosis (TCGA data; TMPRSS2-ERG-positive samples). All the genes have passed the following thresholds: DESeq *p*-value < 0.01, Mann–Whitney *p*-value < 0.05, average expression-level fold change > 25%. Dot size indicates *k*/*n* ratio (“gene ratio”), where *k* is the number of genes participating in the current KEGG pathway (within top-40, top-80, or other top lists), and *n* is the number of genes (also within top-40, top-80, or other top lists) annotated as participants of any KEGG pathway (these numbers are provided in the brackets in the bottom of the figure). Dot color indicates the enrichment test FDR (Fisher’s exact test).

**Figure 6:**
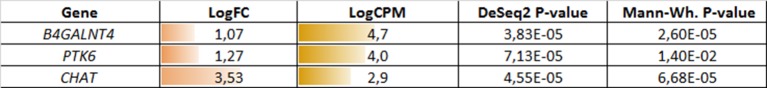
Potential markers of unfavorable prognosis in lymph node-negative LAPC involved in the enriched KEGG pathways (TCGA data, TMPRSS2-ERG-positive samples). LogFC—trimmed binary logarithm of average expression-level fold change (between the groups of favorable and unfavorable prognosis); LogCPM—binary logarithm of CPM.

In a similar manner, we performed bioinformatic analysis for the TCGA sample groups characterized by the expression of the fusion transcript *TMPRSS2-ERG*. Comparative analysis between the groups with favorable and unfavorable prognosis revealed 127 DEGs (**Supplementary Table S3**). We identified 61 genes that were characterized by Mann–Whitney *p* < 0.05 (**Figure 4**). The KEGG pathway analysis revealed several over-represented ones; these are present in **Figure 5** and Supplementary **Table S6**. **Figure 6** represents the genes involved in the enriched KEGG pathways that can be potential marker in lymph node-negative LAPC TMPRSS2-ERG subtype.

### Bioinformatic Analysis of the Russian Patient Cohort

**Figure 7:**
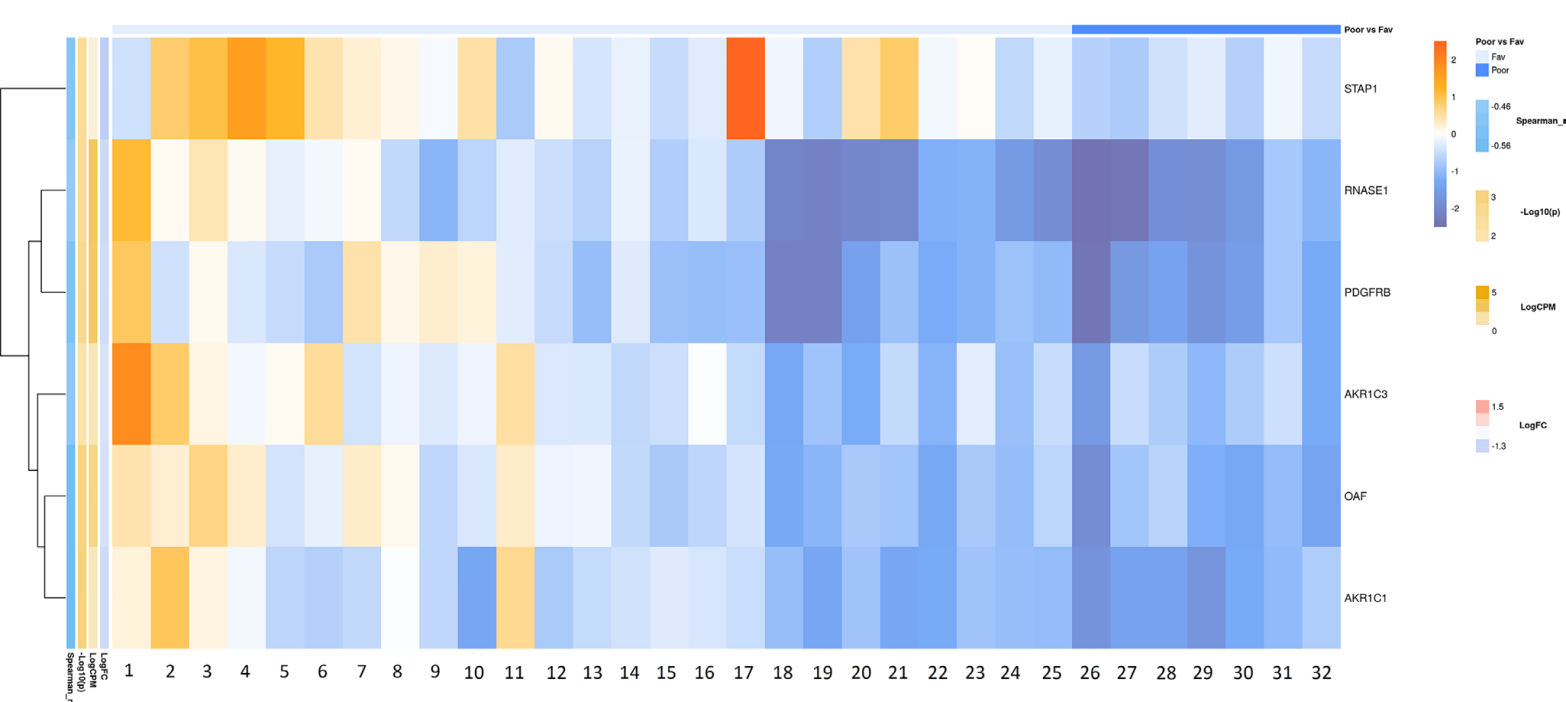
Heatmap demonstrating log relative expression level of top differentially expressed genes between the groups of adverse and favorable prognosis (Russian patient cohort). Cell colors (blue-white-orange gradient) correspond the binary logarithm of the ratio of the expression level in a current sample to the average level across all the samples (per each gene). Blue - expression level is below the average, orange - above the average. Spearman_r—Spearman’s rank correlation coefficient. -Log10(p) is the negative logarithm of DESeq’s *p*-value. LogCPM—average binary logarithm of read counts per million (CPM). LogFC—binary logarithm of expression-level fold change (sample with adverse prognosis compared to favorable prognosis).

**Figure 8:**
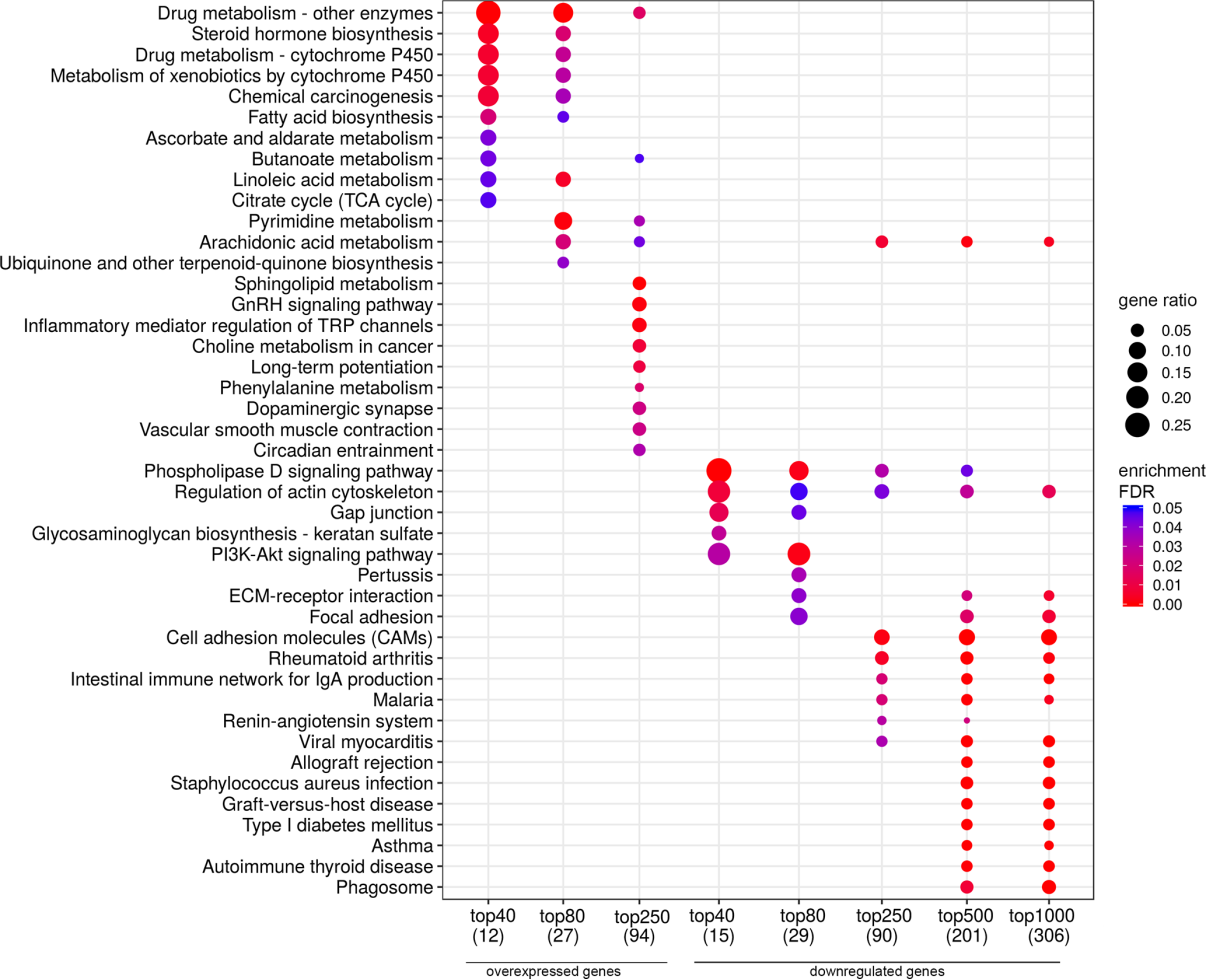
Dotplot showing the results of KEGG pathways enrichment analyses performed for top-40, 80, 250, 500, and 1,000 differentially expressed genes (either overexpressed or downregulated) between the groups of adverse and favorable prognosis (Russian patient cohort). All the genes have passed the following thresholds: DESeq *p*-value < 0.01, Mann–Whitney *p*-value < 0.05, average expression-level fold change > 25%. Dot size indicates *k*/*n* ratio (“gene ratio”), where *k* is the number of genes participating in the current KEGG pathway (within top-40, top-80 or other top lists), and *n* is the number of genes (also within top-40, top-80, or other top lists) annotated as participants of any KEGG pathway (these numbers are provided in the brackets in the bottom of the figure). Dot color indicates the enrichment test FDR (Fisher’s exact test).

**Figure 9:**
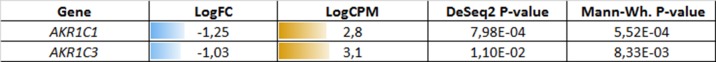
Potential markers of unfavorable prognosis in lymph node-negative LAPC involved in the enriched KEGG pathways (Russian patient cohort). LogFC—trimmed binary logarithm of average expression-level fold change (between the groups of favorable and unfavorable prognosis); LogCPM—binary logarithm of read counts per million (CPM).

According to the results of bioinformatic analysis of lymph node-negative LAPC samples from the Russian patient cohort, we identified 63 DEGs with the expression-level changes of 2-fold or greater between the prognostic groups studied that are passed DESeq2 threshold (**Supplementary Table S4**). We then used Mann–Whitney test and revealed six potential markers of unfavorable prognosis (**Figure 7**). We performed KEGG pathway enrichment analysis for 81 DEGs identified (**Figure 8**, **Supplementary Table S7**). Several enriched KEGG pathways were identified: phospholipase D signaling pathway, GnRH signaling pathway, cell adhesion molecules (CAMs), allograft rejection, and some metabolic pathways (including sphingolipid metabolism) were found to be correlated with the tumor prognosis. These pathways included two genes (*AKR1C1* and *AKR1C3*) with Mann–Whitney *p* < 0.05 (**Figure 9**).

## Discussion

Prostate cancer, an age-dependent disease, is caused by alterations in various molecular genetic mechanisms, including mutations and epigenetic changes followed by perturbations in cell signaling and metabolic pathways. Genes, which participate in these pathways, can serve as either diagnostic or prognostic biomarkers.

Our study describes the results of transcriptome profiling of locally advanced lymph node-negative prostate cancer. We have identified several genes that are potentially associated with the unfavorable prognosis of the tumors. The study can be divided into four parts. We first analyzed RNA-Seq data of lymph node-negative LAPC patients from both the TCGA and Russian cohort. Then, we separately studied the tumors of most common PC molecular subtype carrying fusion transcript *TMPRSS2-ERG* across the patients grouped by favorable and unfavorable prognosis.

In the first part of the study, we examined the TCGA data and identified 60 genes that were differentially expressed between favorable and unfavorable prognosis groups (with Mann–Whitney *p* < 0.05) and could be potential prognostic markers. Three genes (*B4GALNT4*, *PTK6*, and *CHAT*) were involved in the enriched KEGG pathways.

*B4GALNT4* encodes for a beta-1,4-N-acetyl-galactosaminyltransferase 4. It was found to be upregulated in prostate, breast, and lung cancers (Fukushima et al., 2010; Potapenko et al., 2010; Zhang et al., 2017). The encoded protein is involved in the glycan metabolism and seems to be responsible for beta-N-acetylgalactosaminylation of a prostate-specific antigen (PSA) during prostate carcinogenesis (Fukushima et al., 2010).

*PTK6* encodes for a cytoplasmic nonreceptor tyrosine kinase that may act as an intracellular signal transducer in epithelial tissues. Overexpression of *PTK6* in mammary epithelial cells leads to sensitization of the cells to epidermal growth factor and results in a partially transformed phenotype (GeneCards). *PTK6* is highly expressed in multiple tumor types, including prostate, ovarian, and breast cancers, and participates in the regulation of tumor cell proliferation, migration, and survival (Ito et al., 2017). The encoded protein represents a prominent therapeutic target (Park et al., 2015). In prostate cancer, *PTK6* contributes to increased phosphorylation and activation of its substrates, such as AKT serine/threonine kinase 1 (AKT), CAS scaffolding protein family member 1 (p130CAS), and focal adhesion kinase (FAK), thereby promoting the tumor progression (Zheng and Tyner, 2013).

*CHAT* gene encodes for choline O-acetyltransferase catalyzing the biosynthesis of the neurotransmitter acetylcholine. Acetylcholine is the main mediator in the parasympathetic nervous system providing neuromuscular transmission. It was shown that in the prostate epithelium, CHAT participates in autocrine cholinergic signaling potentially involved in prostate cancer invasion and metastasis (Wang et al., 2015).

The second part of our results refers to lymph node-negative LAPC samples obtained from the Russian patients. Totally, we identified 12 DEGs, potential prognostic markers (passed Mann–Whitney *p* < 0.05 threshold). Two genes from them were involved in the enriched KEGG pathways, the steroid hormone biosynthesis, and arachidonic acid metabolism. As most primary prostate cancers are strongly hormone-dependent, the steroid hormone (including testosterone) biosynthesis pathway plays a pivotal role in the tumor growth, particularly, when androgens are actively synthesized within tumor cells (Nelson et al., 2002; Imamoto et al., 2008; van der Sluis et al., 2012). We have identified two DEGs, *AKR1C1* and *AKR1C3*, encoding for aldo-keto reductase family 1 member C1 and aldo-keto reductase family 1 member C3, respectively. These enzymes participate in the steroid hormone biosynthesis and were shown to be highly expressed in different cancer types (Lewis et al., 2004; Sayagues et al., 2016).

AKR1C1 converts progesterone into its inactive form, a 20-alpha-dihydroxyprogesterone (Nishizawa et al., 2000). Overexpression of *AKR1C1* is associated with cancer progression; however, the mechanism, by which *AKR1C1* promotes metastasis, has not been understood (Ji et al., 2004; Chien et al.; 2009). Several studies suggest a critical role for the canonical function of AKR1C1 in the aggressiveness of hormone-related cancers, which is especially interesting in case of prostate cancer (Ji et al., 2004; Rizner et al., 2006).

AKR1C3, which is also involved in arachidonic acid metabolism, plays a role in the progression of prostate cancer and its resistance to hormone therapy (Adeniji et al., 2013; Chen et al., 2015; Powell et al., 2015). Elevated expression of *AKR1C3* is associated with the progression and aggressiveness of prostate cancer (Stanbrough et al., 2006; Wako et al., 2008).

The third part of our analysis was focused on the identification of prognostic markers in the TMPRSS2-ERG PC molecular subtype based on the TCGA data. The bioinformatic analysis revealed 74 DEGs between favorable and unfavorable prognosis groups with Mann–Whitney *p* < 0.05. Among them, several genes were involved in the enriched KEGG pathways: *ASRGL1*, *MYBPC1*, *RGS11*, *SLC6A14*, *GALNT13*, *ST6GALNAC1*, and *B4GALNT4*.

*ASRGL1* gene encodes for asparaginase–like protein 1 probably belonging to the β-aspartyl peptidase family (Cantor et al., 2009). ASRGL1 catalyzes the hydrolysis of L-asparagine and β-aspartyl dipeptides. In the recent study based on the TCGA data, elevated expression of *ASRGL1* gene was found to be associated with biochemical recurrence of prostate cancer (Chu et al., 2018). On the contrary, decreased expression of ASRGL1 was significantly associated with poor prognosis and reduced disease-specific survival in endometrioid endometrial adenocarcinoma (Edqvist et al., 2015).

*MYBPC1* gene encodes for a member of the myosin-binding protein C family that plays an important role in muscle contraction by recruiting muscle-type creatine kinase to myosin filaments. Increased expression of *MYBPC1* gene was observed in breast cancer (Hu et al., 2014). It is assumed that this gene can take part in the progesterone signaling that is associated with the progression of hormone-associated cancers (Ji et al., 2004).

Regarding the other DEGs, there are many already known cancer-associated ones. Overexpression of *RGS11* gene, encoding for a regulator of G protein signaling 11, promotes cell migration and is associated with advanced stages and aggressiveness of lung adenocarcinoma (Yang et al., 2016). Amino acid transporter, SLC6A14, might represent a possible therapeutic target for pancreatic cancer, and it was found to be upregulated in patient-derived xenografts and primary pancreatic tumors, as well as colorectal cancer (Gupta et al., 2005; Coothankandaswamy et al., 2016).

*ST6GALNAC1* gene encodes for sialyltransferase, which is involved in mucin-type O-glycan biosynthesis. Mucin-type O-glycan is of special interest in the context of carcinogenesis since ectopic expression of mucin-type glycoproteins is a hallmark of epithelial tumors. Different alterations in mucin-type O-glycan pathways are associated with unfavorable prognosis in various tumor types (Byrd and Bresalier, 2004). *ST6GALNAC1* gene is directly activated by androgens and is frequently overexpressed in prostate cancer (Munkley et al., 2015; Munkley et al., 2016). The encoded protein catalyzes the synthesis of the cancer-associated sialyl-Tn antigen (sTn), which is present in a variety of carcinomas and is associated with metastasis and unfavorable prognosis (Pinho et al., 2007). High expression of sTn has been detected in up to half of the high-grade prostate tumors (Myers et al., 1994). It was also found that the expression of *ST6GALNAC1* in prostate cancer cells changes global gene expression profiles toward mesenchymal-like pattern increasing cell mobility and decreasing cell adhesion (Wei et al., 2008). The results of these studies confirm the potential prognostic significance of *ST6GALNAC1* gene for prostate cancer.

The *GALNT13* gene encodes for the polypeptide N-acetylgalactosaminyltransferase 13, which is a member of the GalNAcT family involved in the initiation of O-linked glycosylation of mucins. This gene is expressed in most primary and metastatic epithelial malignant tumors (Wang et al., 1997).

*B4GALNT4* gene was also found to be differentially expressed between favorable and unfavorable prognosis groups in the whole set of LAPC samples from TCGA data (first part of the study). This gene demonstrates great potential prognostic value.

As the results of this study, we identified several genes that can represent potential prognostic markers for LAPC. These genes are participants of several processes associated with the development of various types of cancer; however, none of these genes has an established association with the prognosis of prostate cancer. The results of our research could be used as a basis for the test system development of improving the prognosis, treatment strategy, and management of prostate cancer patients.

## Ethics Statement

The study was approved by The Ethics committee of P.A. Hertzen Moscow Oncology Research Center, Ministry of Health of the Russian Federation. The study was done in accordance with the principles outlined in the Declaration of Helsinki (1964). All patients gave their informed consent for participation in the study.

## Author Contributions

EP, EL, ADK, KN, NV, GE, BA, and AVK conceived and designed the work; EP, AB, KK, AS, DM, and AZ performed the experiments; EL, MK, GK, AAK, and MS analyzed the data; EP, EL, AS, NM, and AVK wrote the manuscript. All authors read and approved the final manuscript.

## Funding

This work and publication costs were funded by the Russian Science Foundation, grant 18-75-10127.

## Conflict of Interest Statement

The authors declare that the research was conducted in the absence of any commercial or financial relationships that could be construed as a potential conflict of interest.
